# Stenosing tenosynovitis with rice bodies formation diagnosed by ultrasound: A case report

**DOI:** 10.1097/MD.0000000000028871

**Published:** 2022-02-18

**Authors:** Lei Ge, Lei Zhang, Libin Lu

**Affiliations:** Department of Emergency, People's Hospital of Rizhao, Jining Medical University, Shandong, China.

**Keywords:** rice bodies, stenosing tenosynovitis, ultrasound

## Abstract

**Rationale:**

Rice bodies are usually found in several nonspecific chronic inflammatory diseases that are symptomatically dominated by primary disease and local compression symptoms. Rice bodies are usually detected by magnetic resonance imaging; however, some remote areas and areas with poor economic conditions do not have access to magnetic resonance imaging examination, which leads to delayed diagnosis of the disease.

**Patient concerns:**

We report the case of a 62-year-old man with pain in the metacarpophalangeal joint of his right middle finger and limited flexion activity of his middle finger.

**Diagnoses:**

The mass was 1 cm, well-circumscribed, soft, and painless. Ultrasound showed stenosing tenosynovitis with rice body formation.

**Interventions:**

The patient underwent tenosynovectomy with synovectomy of the right middle finger tendon sheath under plexus block anesthesia.

**Outcomes:**

No postoperative complications were noted. A 6-month follow-up showed no recurrence. The activity of the patient's middle finger improved significantly.

**Lessons:**

Stenosing tenosynovitis with rice body formation is a very rare condition, and we use ultrasound for diagnosis. Ultrasound is convenient, rapid, inexpensive, and can obtain blood flow information, facilitate disease follow-up, and even allow ultrasound localization in advance for guided needle biopsy.

## 1 Introduction

Rice body synovitis is a rare and nonspecific chronic inflammatory condition.^[1]^ At present, the cause of rice body formation remains unclear. It is most common in rheumatoid arthritis, pulmonary tuberculosis, juvenile arthritis, serum-negative arthritis, osteoarthritis, suppurative arthritis, trauma, chronic bursitis, and other diseases, which can appear in the joints of these patients.^[^2^–^4^]^ In clinical practice, rice bodies in chronic inflammation are mainly found by magnetic resonance imaging (MRI), but color Doppler ultrasound has obvious characteristics of convenience, rapidity, and low price. We report a case of stenosing tenosynovitis with rice body formation using ultrasound and briefly discuss the clinical significance of rice bodies with a literature review.

## 2 Case report

### 2.1 Clinical summary

The patient, male, 62 years’ old, had pain in the metacarpophalangeal joint of his right middle finger with limited activity for >6 months. One month prior, the patient felt that the symptoms were significantly aggravated, and there was a mass at the transverse stria of the palm. The patient was diagnosed with “stenosing tenosynovitis” at the local hospital. He was injected with compound betamethasone injection in the tendon sheath. He felt that his symptoms had improved. In the last 2 weeks, he felt that the mass at the metacarpophalangeal joint had increased rapidly, and the local hospital diagnosed it as “stenosing tenosynovitis.” The patient denied a history of trauma, rheumatoid disease, tuberculosis, or other infectious diseases and denied a family genetic history.

Physical examination revealed a mass in the middle of the transverse palmar crease of the right hand, approximately 1 × 1 cm, with normal local skin temperature, no obvious skin breakdown, soft texture, unclear borders, no local tenderness, significantly limited joint mobility, and contralateral finger was normal.

**Figure 1:**
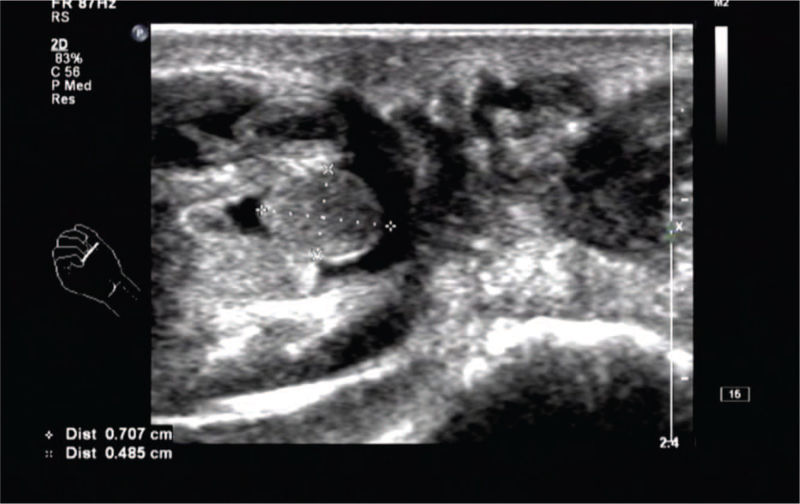
The flexor tendon of the middle finger of the right hand is thickened.

**Figure 2:**
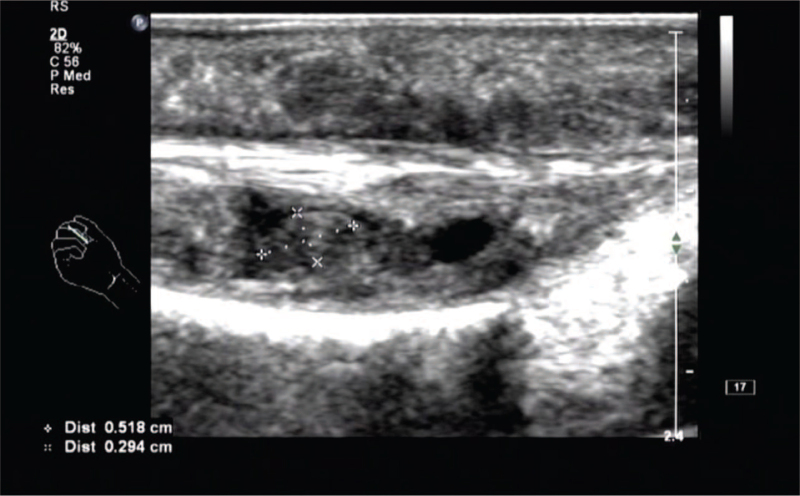
Multiple weak echo masses can be seen in the tendon sheath, with a maximum of about 0.7 × 0.4 cm, oval shape, clear boundary, no obvious blood flow signal in it.

**Figure 3:**
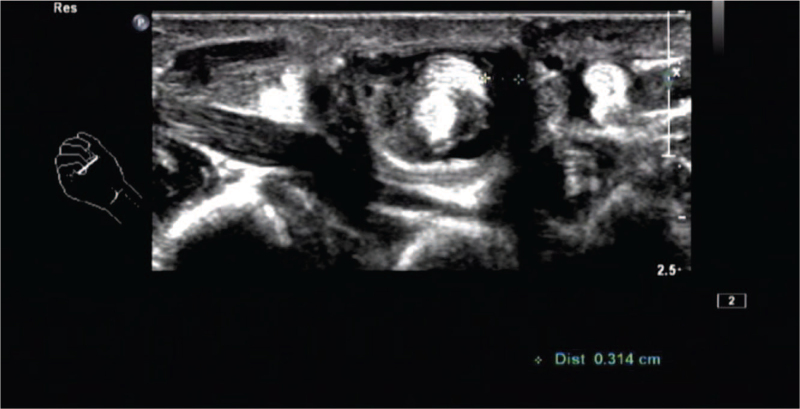
Effusion can be seen in the tendon sheath, about 0.31 cm wide.

Laboratory and ancillary tests: blood routine, liver and kidney function, procalcitonin, erythrocyte sedimentation rate, high-sensitivity C-reactive protein, O-antigen, rheumatoid factor, bone-derived alkaline phosphatase, and autoantibodies were unremarkable, and tuberculosis infection T cell testing was negative. x-Ray showed no obvious abnormalities in the bone mass of the right hand. Ultrasound of the hand showed (Figs. 1–3): the flexor tendons of the right middle finger were thickened, and effusion was seen in the tendon sheath, which was about 0.31 cm in width, blood flow signal was seen in it, and multiple weakly echogenic masses were seen in the tendon sheath, with a maximum size of approximately 0.7 × 0.4 cm. Diagnosis was stenosing tenosynovitis with rice body formation.

**Figure 4:**
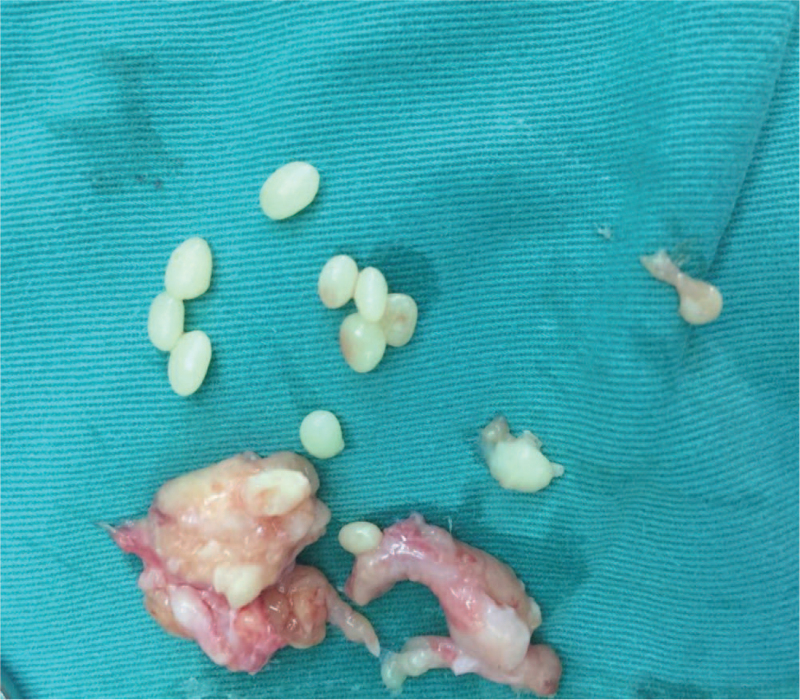
Synovial hyperplasia can be seen in the tendon sheath, and a large number of rice granules can be seen in the synovium.

The patient underwent tenosynovial release with synovectomy of the right middle finger tendon sheath under plexus block anesthesia. Intraoperatively, a *Z*-shaped incision was made along the flexor palmaris tendon of the right hand, and synovial hyperplasia of the tendon pulley was surrounded by white granular-like tissue. Synovial hyperplasia was observed in the tendon sheath, and a large number of rice bodies were observed in the synovium (Fig. 4).

The patient's joint mobility improved significantly 1 month postoperatively and was largely pain-free. At the follow-up visit in March after the operation, the patient's symptoms were better than those before the operation, and the movement of the middle finger was significantly improved.

### 2.2 Pathological findings

**Figure 5:**
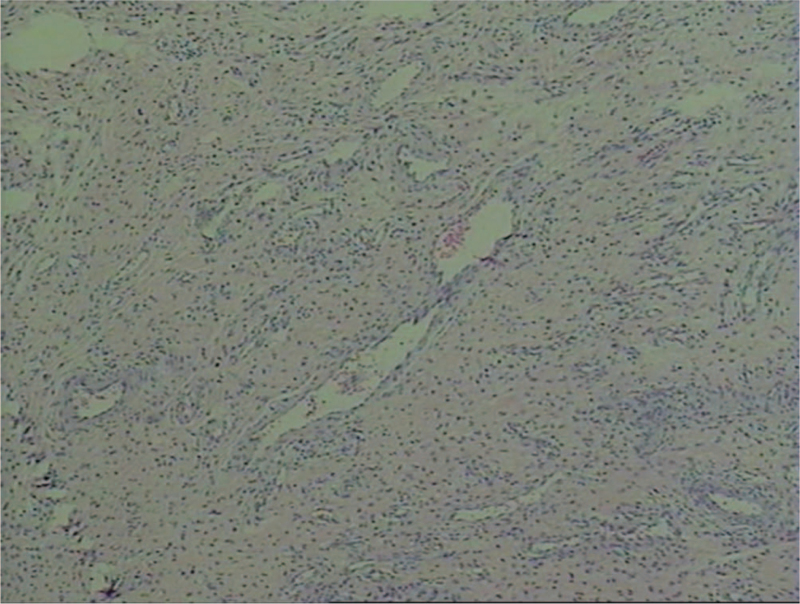
Proliferative synovial tissue showed significant acute and chronic inflammation with infarction and multinucleated giant cell reaction. Several infarct nodules were seen.

Pathological examination (Fig. 5): a large number of multinucleated giant cells invaded the hyperplastic synovial tissue, which presented with acute and chronic inflammatory infarcts, and several infarct nodules were observed, consistent with nonspecific chronic synovial changes.

## 3 Discussion

Although rice body-related diseases have been reported one after another clinically, the specific mechanism of rice body formation remains unclear. Earlier studies using histochemistry, immunofluorescence, scanning, and transmission electron microscopy of rice bodies revealed that they were mainly composed of fibrin and a small amount of collagen, as well as cells, blood vessels, and other structures, and were associated with the synovial membrane.^[5]^ Rice bodies may be formed by microinfarcts in diseased synovial tissue.^[6]^ In other studies, scholars have proposed different insights into the formation mechanisms of rice bodies. The formation of rice bodies by an extra-articular foreign body reaction has been reported by Muirhead et al. Rice bodies in chronic tenosynovitis caused by foreign bodies are mainly composed of fibroblasts, cellulose, and collagen, and are somewhat different from the structure of rice bodies of synovial origin. Studies have speculated that rice bodies may be associated with fibroblasts encapsulated and activated by cellulose, which, in turn, produces collagen.^[7]^ Some scholars have found a large number of rice bodies in the capsule of patients with chronic infection after hip arthroplasty, which indicates that rice bodies can also form after chronic infection.^[8]^ It can be seen that rice bodies like structures can form in different diseases, and there are some differences between the formation mechanisms and components of these rice bodies. In our case, rice body synovitis in the tendon sheath of the flexor tendons of the right hand was rare, and the patient had no history of rheumatoid arthritis, tuberculosis, or other diseases, which were considered primary lesions.

Magnetic resonance imaging (MRI) is a common examination method used for the diagnosis of rice body synovitis. The specific manifestations are a nodular slightly high signal on T2WI and fat suppression weighted, low signal on T1WI, and no enhancement after enhancement. However, compared to MRI, ultrasound examination is convenient, fast, cheap, and can obtain blood flow information, which is simpler and applicable in the follow-up of the disease, postoperative recurrence, and guiding puncture biopsy.^[9]^ Therefore, in this case, we found that the patient did not undergo MRI, which is conducive to reducing the hospitalization cost of the patient, however we were still able to establish the presence of rice bodies in the tendon sheath by ultrasound.

The preoperative ultrasonic diagnosis of tenosynovitis with rice bodies in this case was consistent with the postoperative pathological diagnosis. Postoperative pathology showed inflammatory cell infiltration and fibrous tissue wrapping at the edge of the rice bodies, which may explain the appearance of white rice bodies. Sivaloganathan observed that under a light microscope, they appear as multiple inflammatory free bodies in the articular cavity of rice granulosa bursitis, with eosinophils in the center and infiltration of plasma and lymphocytes around and the outer edge isa fibrin like substance.^[10]^ Several studies have shown that chronic inflammation such as tuberculosis, osteoarthritis, rheumatoid arthritis, and fungal infection can lead to the formation of rice bodies.^[^11^,^^12^^]^ Combined with the medical history, serological examination, and postoperative pathological examination, the patient had no chronic diseases, such as tuberculosis, rheumatism, infection, or osteoarthritis. The patient was diagnosed with chronic stenosing tenosynovitis, resulting in the formation of rice bodies.

In conclusion, the causes of rice body formation are diverse, and are a common pathological manifestation of many diseases. During the treatment of granulation-related diseases, attention should be paid to the cause of the disease. Immunological and infectious diseases must be excluded. It is of great significance to improve the therapeutic effects and prevent recurrence. In terms of treatment, rice body-related diseases tend to be benign, and focal resection is performed through open surgery or arthroscopic minimally invasive surgery. For related basic diseases, we should also provide corresponding anti-inflammatory, anti-tuberculosis, or anti-infection treatments to improve the treatment effect and reduce the chance of recurrence.

## Author contributions

**Conceptualization:** lei ge.

**Data curation:** lei ge, Libin Lu.

**Formal analysis:** Lei Zhang.

**Investigation:** Lei Zhang.

**Resources:** Libin Lu.

**Software:** Lei Zhang.

**Writing – original draft:** lei ge.

**Writing – review & editing:** Libin Lu.
